# The Foreign Journals: Statistics

**Published:** 1840-07

**Authors:** 


### STATISTICS.

*Revaccination in the Prussian Army in* 1839. By Dr. Lohmeyer,

| | | |
|---|---|---|
| Total revaccinated | | 41,481 |
| Of this number there were *distinct* marks of previous vaccination in | 33,225 | |
| *indistinct* in | 5,889 | |
| none in | 2,367 | |
| The course of the disease was regular in | | 19,249 |
| irregular in | | 8,534 |
| And no effect followed in | | 13,698 |
| Out of the number that remained unaffected in the first instance, the operation was repeated with effect in | 2,105 | |
| without effect in | 7,886 | |

Out of the number revaccinated, in whom the disease ran a regular course (19,249), there were produced perfect pustules as follows:

| | |
|---|---|
| 1 to 5 pustules in | 8,762 |
| 6 .. 10 | 5,650 |
| 11 .. 20 | 4,095 |
| 21 .. 30 | 742 |

Of those revaccinated with effect in 1839 and previously, there were attacked in the course of the same year,

| | |
|---|---|
| With varicella | 18 |
| With varioloid disease | 7 |
| With smallpox | 0 |

The revaccinations, as in former years, were made partly from arm to arm, and partly (in the beginning of the process) from dried lymph. In the operations from arm to arm, the lymph was taken partly from fresh-vaccinated children, partly from adults vaccinated or revaccinated. The results, as in former years, were so far similar in the two latter cases, that true pustules equally followed from either source; but it appeared from the testimony of several of the surgeons that there ensued a more powerful reaction with considerable fever in the stage of maturation, from the insertion of the lymph from adults than from children. The inoculations from dried lymph often failed.

As had also been formerly observed, the disease was often remarkably well characterized in individuals in whom the scars of previous vaccination were strongly marked, while it often failed, and after repeated trials, in others who showed no trace of a former disease.

By way of experiment, many individuals, in whom there existed more or less distinct scars from *smallpox*, were vaccinated, and frequently in these there ensued perfectly good pustules both as to appearance and course.

*Berl. Medic. Zeitung.* *April* 22, 1840.

